# Computational On-Chip Imaging of Nanoparticles and Biomolecules using Ultraviolet Light

**DOI:** 10.1038/srep44157

**Published:** 2017-03-09

**Authors:** Mustafa Ugur Daloglu, Aniruddha Ray, Zoltan Gorocs, Matthew Xiong, Ravinder Malik, Gal Bitan, Euan McLeod, Aydogan Ozcan

**Affiliations:** 1 Electrical Engineering Department, University of California, Los Angeles, CA, 90095, USA; 2 Bioengineering Department, University of California, Los Angeles, CA, 90095, USA; 3 California NanoSystems Institute (CNSI), University of California, Los Angeles, CA, 90095, USA; 4 Department of Neurology, David Geffen School of Medicine, University of California, Los Angeles, CA, 90095, USA; 5 Brain Research Institute, University of California, Los Angeles, CA, 90095, USA; 6 Molecular Biology Institute, University of California, Los Angeles, CA, 90095, USA; 7 College of Optical Sciences, University of Arizona, Tucson, AZ 85721, USA; 8 Department of Surgery, David Geffen School of Medicine, University of California, Los Angeles, CA, 90095, USA

## Abstract

Significant progress in characterization of nanoparticles and biomolecules was enabled by the development of advanced imaging equipment with extreme spatial-resolution and sensitivity. To perform some of these analyses outside of well-resourced laboratories, it is necessary to create robust and cost-effective alternatives to existing high-end laboratory-bound imaging and sensing equipment. Towards this aim, we have designed a holographic on-chip microscope operating at an ultraviolet illumination wavelength (UV) of 266 nm. The increased forward scattering from nanoscale objects at this short wavelength has enabled us to detect individual sub-30 nm nanoparticles over a large field-of-view of >16 mm^2^ using an on-chip imaging platform, where the sample is placed at ≤0.5 mm away from the active area of an opto-electronic sensor-array, without any lenses in between. The strong absorption of this UV wavelength by biomolecules including nucleic acids and proteins has further enabled high-contrast imaging of nanoscopic aggregates of biomolecules, e.g., of enzyme Cu/Zn-superoxide dismutase, abnormal aggregation of which is linked to amyotrophic lateral sclerosis (ALS) - a fatal neurodegenerative disease. This UV-based wide-field computational imaging platform could be valuable for numerous applications in biomedical sciences and environmental monitoring, including disease diagnostics, viral load measurements as well as air- and water-quality assessment.

Humankind has reached unprecedented control over the nanoworld by creating nanoscale objects and manipulating them for a wide spectrum of applications extending from electronics to biomedical sciences. As in many nano applications, significant progress in the characterization of nanoparticles^1^,^2^ and the investigation of nanoscale biomolecules and supramolecular structures, such as viruses^3^,^4^,^5^, protein aggregates^6^,^7^ and exosomes^8^,^9^, was enabled by the development of powerful imaging and sensing tools including scanning and transmission electron microscopy (SEM and TEM)^3^,^4^,^5^, atomic force microscopy (AFM)^10^,^11^, stimulated emission depletion (STED) microscopy^12^,^13^, localization based super-resolution microscopy^14^,^15^, and near-field scanning optical microscopy (NSOM)^16^,^17^ among many others.

These imaging and sensing techniques and instruments have played a critical role in expanding the boundaries of our knowledge. In order to fully benefit from these technological advances and extend our measurement capabilities outside the laboratory, it is necessary to develop more accessible alternatives to these laboratory-bound devices^18^. Towards this direction, on-chip holographic microscopy^19^,^20^,^21^,^22^,^23^ offers a robust, cost-effective and high-throughput alternative to some of the existing imaging and sensing techniques. In an on-chip computational microscope, the sample is placed very close (i.e., ≤0.5–1 mm) to an opto-electronic image sensor chip without any imaging lenses in between. As a result, the imaging field-of-view (FOV) is equal to the active area of the sensor chip and is decoupled from spatial resolution, unlike a lens-based microscope design, where there is a fundamental trade-off between the two^22^. This on-chip imaging design has unit magnification and therefore its spatial resolution is limited by the pixel pitch of the imager chip. This limitation can be overcome to push the resolution of an on-chip microscope to the diffraction limit by using pixel super-resolution approaches^24^,^25^, e.g., by using an array of light sources to synthesize a high resolution image of the specimen from sub-pixel shifted versions of its hologram. Recently, this platform was also converted into a powerful nanoscale imaging device by self-assembly of polymer nano-droplets, or nanolenses, around the target particles to significantly enhance their optical signatures^26^,^27^,^28^,^29^, enabling the detection of sub-40 nm particles over a FOV of >20–30 mm^2^ 27,28,29.

This particle detection limit is mainly determined by the signal-to-noise ratio (SNR) of the on-chip imaging system and can be further improved by either increasing the optical signal received from the nanoparticles (or nanoparticle-nanolens complexes) or decreasing the background noise level across the image sensor. By using more advanced image sensors with active cooling capabilities, for example, the dark noise could be reduced, improving the SNR of the imaging system^30^. The SNR could also be improved by boosting the signal received from the target objects, which is strongly dependent on the amount of photons scattered towards the sensor with respect to the background illumination. This relative scattering power of nanoparticles, or the forward scatter radar cross section (RCS), is dependent on the illumination wavelength and could be increased with shorter wavelengths^31^,^32^.

Another way to boost the optical signatures of the target particles is through absorption. Most biomolecules have absorption peaks at certain optical frequencies; for example proteins and nucleic acids have strong absorption lines in the ultraviolet (UV) wavelengths^33^,^34^. This property of UV light as an inherent contrast agent through absorption has been used in conventional brightfield microscopy^34^,^35^ for imaging of e.g., cancer tissue samples^36^, live cells^37^ and protein microcrystals that are >2–5 μm in size^38^. Nucleic acid and protein mass mapping within cells have also been enabled through UV microscopy, by capturing transmission images at different UV wavelengths^39^,^40^,^41^. Furthermore, UV light has been used to selectively excite nucleic acids for photoacoustic microscopy, highlighting the nuclei in the tissue images^42^ and to increase the resolution for darkfield imaging of larger viral particles^43^. Coherent UV light sources have been used in holographic imaging for increased resolution^44^ and sensitivity^45^, but these techniques were also limited in throughput and FOV due to the use of imaging lenses and a large magnification factor.

Combining these intrinsic properties of UV light with the unique capabilities of on-chip holographic imaging, we have designed and built an on-chip computational microscope operating at a UV wavelength of 266 nm. The increased scattering from nanoparticles due to the shorter wavelength enabled us to surpass the previous detection limit and image sub-30 nm nanoparticles, with a smallest detectable particle diameter of ~26 nm. Furthermore, we demonstrated the application of this method to imaging aggregates of Cu/Zn-superoxide dismutase (SOD1) with significantly improved contrast relative to other on-chip imaging platforms that use visible illumination wavelengths. Aggregation of SOD1 has been linked to the fatal disease, amyotrophic lateral sclerosis (ALS), caused by the death of motor neurons^46^,^47^,^48^. This unique UV-based wide-field computational imaging platform would be valuable for numerous applications in biomedical sciences and environmental monitoring, such as disease diagnostics, viral load measurements, as well as air- and water-quality monitoring.

## Results and Discussion

**Figure 1:**
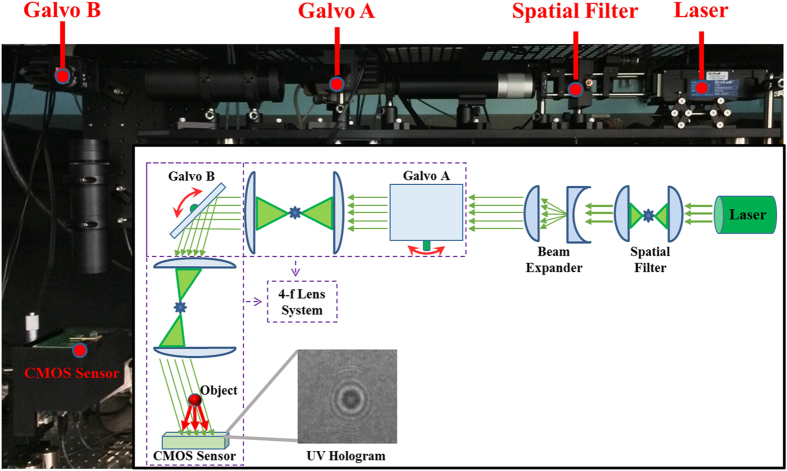
The holographic on-chip imaging platform operating at 266 nm illumination wavelength. The laser beam is first spatially filtered and then expanded to cover the full CMOS imager active area. The object is directly placed on top of the image sensor, which records in-line holograms of the object across an imaging FOV of >16 mm^2^. A pair of scanning galvo mirrors controls the angle of incidence to shift the holograms across the image sensor active area, while a pair of 4-f lens systems helps to keep the beam fixed on the sample plane. Note that that there are no imaging lenses between the sample and the image sensor chip, which are separated from each other by ≤0.5 mm.

Our holographic on-chip imaging platform consists of a 4^th^ harmonic Nd:YAG laser generating 266 nm light, a custom-designed free-space light delivery system composed of multiple UV grade lenses and scanning galvo mirrors, and a CMOS image sensor chip (Fig. 1). A uniform laser beam profile is generated using a spatial filter, by focusing the beam through a 25 μm pinhole and then expanding it by a factor of 10 to have a large beam diameter covering the full area of the CMOS image sensor chip, which is also equivalent to our imaging FOV (i.e., FOV = 16.45 mm^2^). Passing through a system of scanning galvo mirrors and UV lenses, the UV light is then guided towards the sample that is placed directly on top of the sensor chip (i.e., ~300–500 μm above the active area). The direct interference of the background illumination light with the scattered object light creates in-line holograms of the specimen, which are then recorded/digitized by the image sensor chip. The 3D information of the sample is encoded within these holographic interference patterns, which can be extracted by digitally back propagating the holograms to the sample plane revealing microscopic reconstructions, both as amplitude and phase images, of the target objects^19^,^22^,^49^,^50^.

The resolution of the reconstructed images in our on-chip microscope is mainly limited by the pixel pitch (1.12 μm) of the image sensor chip, which sets a physical limit on the spatial sampling of the holographic fringes. It is possible to bypass this limit by using pixel super-resolution (pixel SR) techniques that combine multiple low resolution images of the object, each shifted by sub-pixel amounts with respect to each other, to generate a high resolution image with a smaller effective pixel pitch, thus a higher spatial sampling rate^24^,^51^. To laterally shift the holograms on the image sensor, we adjust the angle of incidence of the light on the sample by tuning the angular positions of the two scanning galvo mirrors that are placed in orthogonal tilting axes. The 4-f lens systems between the first and second galvo mirrors and between the second galvo mirror and the sample help to keep the beam location fixed on the sample plane while also tuning the angle of incidence, which is important to keep the full image sensor area illuminated as well as to shift the holograms in both lateral directions across the image sensor plane (Fig. 1). This imaging set-up enables us to fully benefit from the unique properties of on-chip imaging in the UV part of the spectrum, where the imaging FOV is decoupled from the spatial resolution and is scalable with the active area of the image sensor, unlike lens-based microscopy tools. It is important to note that by on-chip, we mean that *there are no imaging lenses between the sample and the image sensor chip* and the lenses used in our free space light delivery system do not limit our FOV.

**Figure 2:**
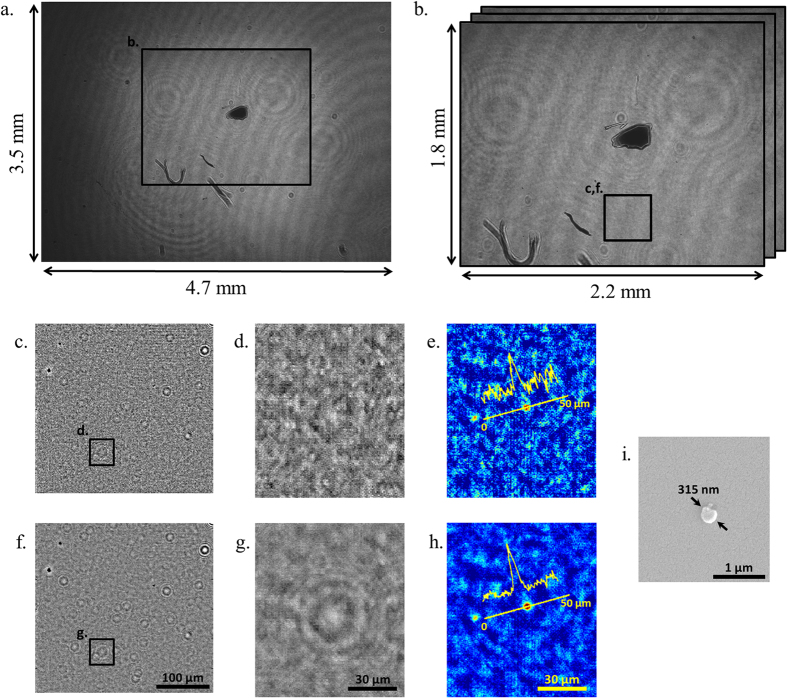
(**a**) A full FOV image (FOV > 16 mm^2^) captured by the UV on-chip imaging platform. (**b**) A zoomed in image taken from (**a**). (**c**) and (**f**) Super-resolved holographic image generated by only the first 196 sub-pixel shifted frames (no circular averaging) and by all the 556 images (196 sub-pixel shifted and 360 circularly shifted), respectively. (**d**) and (**g**) A zoomed-in comparison of the super-resolved hologram without and with circular averaging, respectively. The background noise is significantly reduced and the in-line hologram of a particle is more visible with the circular averaging. (**e**) and (**h**) The phase reconstruction of both of the high-resolution holograms shown in (**d**) and (**g**), respectively. As visible from the image profile across the target, circular averaging helps suppress the background noise considerably. The target in this example was a 315-nm nanoparticle, imaged after nanolens deposition^28^,^29^. (**i**) The SEM image of the target nanoparticle in (**e**) and (**h**).

There are, however, additional steps to be taken when using a laser light source with very high temporal and spatial coherence in holographic on-chip imaging, which give rise to strong speckle noise and multiple reflection related interference artifacts. To suppress both the speckle noise and the background interference fringes originating from various surfaces of the UV illumination system, we take advantage of the pixel SR framework by capturing a larger number of low resolution images that is redundant for image resolution, but is very effective in smearing out such coherence-related noise terms. Because these noise sources are at different optical planes compared to the sample plane on the CMOS imager, the noise grains and fringes shift by significantly different amounts, and sometimes in opposite directions, compared to the shift of the in-line holograms of the target objects located on the sample plane. When our pixel SR algorithm^52^,^53^ locks to the target object holograms, only the holographic interference signal corresponding to the object plane is correctly overlapped and added, while all the noise terms from other planes are spatially smeared out (Fig. 2). In our experiments, a total of 556 low resolution images were used to digitally create a noise-suppressed, high-resolution (i.e., super-resolved) holographic image, where 196 of these 556 images contained sub-pixel shifts of ~0.16 μm on a rectangular grid to improve the resolution and the remaining 360 images were shifted on a circular grid (i.e., composed of 12 concentric circles with a radial spacing of ~0.3 μm and 30 images per circle with equal angular intervals) to further suppress the unwanted noise terms. As illustrated in Fig. 2, this additional circular averaging step in pixel SR plays a significant role in cleaning out various spatial and temporal coherence related noise artifacts in our super-resolved holograms and their reconstructions.

It should be noted that a typical set-up for holographic on-chip microscopy in the visible part of the spectrum is considerably simpler^49^,^54^ compared to the UV part of the spectrum, because visible LEDs cost-effectively provide uniform and sufficiently powerful illumination covering the entire active area of the image sensor chip, while also rejecting the above described coherence-induced noise terms by engineering temporal and spatial coherence of the source, through e.g., spectral filtering (if needed), spatial filtering or simple free-space propagation. As high-power UV LEDs with decent output beam profiles become available, similar approaches can also be used to engineer the temporal and spatial coherence of the UV illumination and further simplify the illumination front-end of our current UV microscopy set-up (Fig. 1).

**Figure 3:**
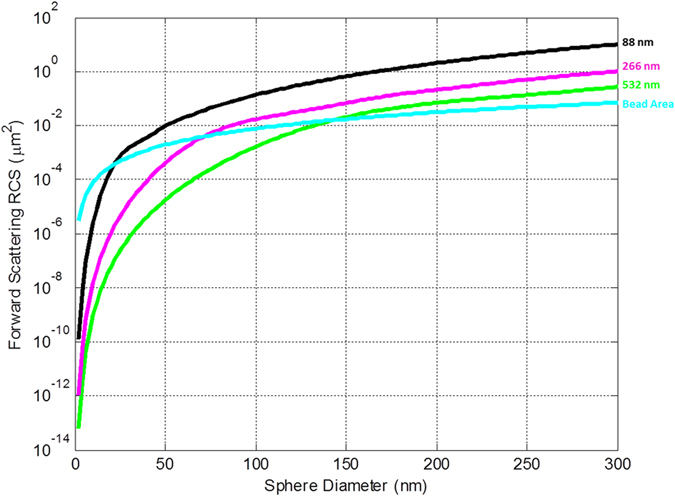
The forward scattering RCS of a perfect electric conductor (PEC) nanosphere under different optical wavelengths (color coded) as a function of its diameter. Based on these RCS values, a detection limit of 250 nm under 532-nm illumination is equivalent to a ~180-nm particle diameter under 266-nm illumination, assuming that the sensor performance and noise levels remain the same. These detection-size limits are significantly improved using self-assembled nanolenses, as illustrated in Fig. 6, enabling the detection of e.g., a ~26 nm particle.

To provide a further comparison between visible and UV illumination and their detection limits, we performed a radar cross section (RCS)^31^,^55^,^56^ analysis as illustrated in Fig. 3. A holographic on-chip microscope using green illumination (e.g., ~532 nm) and an off-the-shelf standard CMOS image sensor (kept at room temperature) is practically limited to a particle size of ~250 nm as the detection limit^50^, which can be further reduced to ~40–50 nm using self-assembled nanolenses^28^,^29^. Assuming that the sensor performance and other noise parameters remain the same, we used RCS analysis to get an estimate on the improvement of this practical detection limit at shorter wavelengths (Fig. 3). Based on this analysis, the forward scattering RCS of a 250-nm diameter particle illuminated with 532-nm light is approximately equivalent to the RCS of ~180-nm bead under 266-nm illumination. This suggests that the practical detection limit should be close to 180 nm with the UV imaging platform under similar circumstances (and without the use of self-assembled nanolenses).

**Figure 4:**
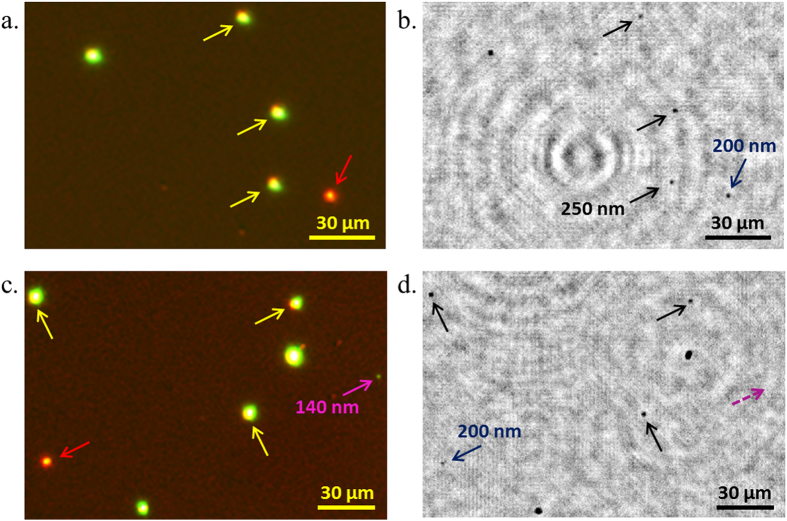
(**a**) and (**c**) Fluorescence microscopy images of green-fluorescent 250-nm beads (yellow arrows), red-fluorescent 200-nm beads (red arrows) and a green-fluorescent 140-nm bead (purple arrow). (**b**) and (**d**) The corresponding amplitude reconstructions from the UV on-chip images with 250-nm beads (black arrows) and 200-nm beads (navy-blue arrows). The 140-nm bead is not visible in the amplitude reconstruction (dashed purple arrow).

To confirm our predictions shown in Fig. 3, the detection performance of the UV on-chip imaging platform was first tested with polystyrene bead samples, *without* nanolens deposition. A bead suspension was prepared by mixing green-fluorescent 140-nm, red-fluorescent 200-nm and green-fluorescent 250-nm polystyrene beads and depositing them onto fused silica slides. The fluorescence of these particles was utilized to cross-validate our on-chip imaging results against a benchtop standard fluorescence microscope. These samples were then imaged with our UV platform, enabling the detection of 250-nm and 200-nm beads, which were independently verified using fluorescence microscopy (Fig. 4). 140-nm beads, however, could not be detected using the UV on-chip microscope, in agreement with the RCS analysis reported in Fig. 3.

The above discussed particle size detection limit could be significantly decreased by depositing polymer nanolenses around target particles through vapor condensation and self-assembly, which enhance the holographic signatures of nanoscale objects^27^,^28^,^29^. This requires an additional step following the capture of the nanoparticles on the surface of the fused-silica substrate, which is exposed to polyethylene glycol (PEG) vapor for 1–5 minutes. Formation of the nanolenses is continuously monitored by recording their holographic signatures using our on-chip microscope. Note that by the time the PEG vapor is exposed to the substrate, it cools down to ≤40 °C and therefore does not damage the sample. Monitoring the temporal evolution of these holographic signatures enables determining the optimal deposition time for achieving the best signal-to-noise ratio. This entire process, including the self-assembly of the nanolenses and their continuous imaging, can be implemented using a compact and field-portable on-chip imaging system^29^. After the vapor deposition of nanolenses around the target particles is completed, the sample is imaged using the UV on-chip imaging platform by capturing 556 frames for pixel SR with circular averaging. Even using a modest and relatively old read-out circuitry, it is possible to acquire all these frames within 15 minutes by decreasing the frame readout size by a factor of 4 (i.e., ~4 mm^2^ FOV, Fig. 2b), which is fast enough to maintain nanolens stability throughout the entire image acquisition.

**Figure 5:**
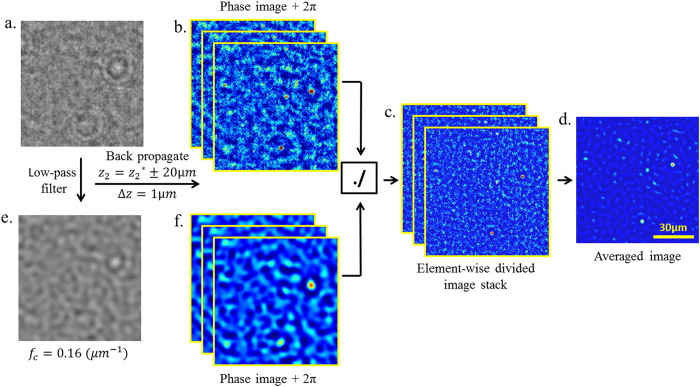
(**a**) and (**e**) High resolution raw image after pixel SR with circular averaging and its low-pass filtered version, respectively. (**b**) and (**f**) Both the original and the low-pass filtered raw frames are back propagated over a small depth neighborhood (e.g., ±20 *μm*) around the optimal reconstruction height (z_2_^*^) and the corresponding phase images are extracted. The reconstructions corresponding to the low-pass filtered images only contain features from the background. (**c**) Each original phase reconstruction from the stack in (**b**) is element-wise divided by its low-pass filtered phase reconstruction from the same height in (**f**), after adding 2π radians to each image. The resulting image stack is a background compensated version of (**b**), only containing higher frequency noise grains. (**d**) Averaging the background compensated stack in (**c**) further suppresses the noise grains.

For the detection of smaller nanoparticles using UV imaging and self-assembled nanolenses, additional digital filtering and processing steps are necessary to further suppress the background noise in the high-resolution raw image obtained after pixel SR with circular averaging (Fig. 5a). To this end, the first step is to compensate for the lower frequency background that is present in the phase reconstruction, by dividing the original phase image (Fig. 5b) with the background phase image itself (Fig. 5f). To extract this background image, the high resolution raw image is first low-pass filtered such that the holograms of the nanoparticles are removed, only leaving the interference patterns corresponding to larger dust particles and the low-frequency portion of the background noise (Fig. 5e), which is then back propagated and the background phase image is extracted. This background image serves as an intensity map of the low-frequency modulation, which is used to suppress or compensate this background through element-wise image division (which we refer to as background compensation). The resulting image (after the element-wise image division, Fig. 5c) is cleaned from the low-frequency background, revealing smaller particles that might have been otherwise buried under the low-frequency modulation of the background or larger particles. Note that to avoid divide-by-zero error, we add 2π to each phase image before the division step.

**Figure 6:**
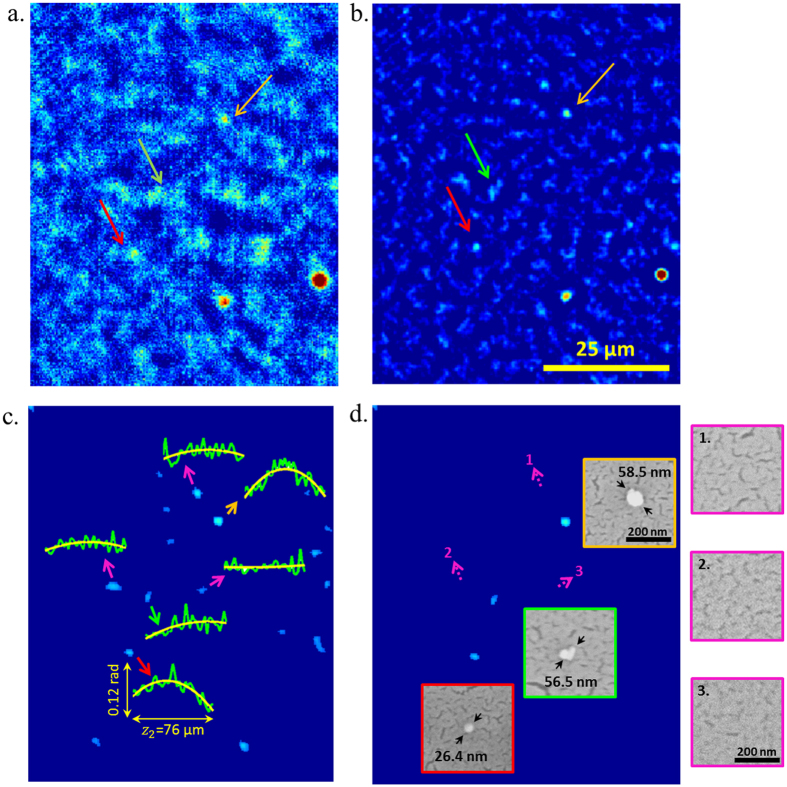
(**a**) Phase reconstruction of a holographic raw image after pixel SR and circular averaging. (**b**) The same FOV after background compensation and averaging over different reconstruction heights (see Fig. 5). Note that the two larger particles (>150 nm), appearing red in the bottom-right corner, are digitally peeled^29^ to highlight the smaller particles in (**c**), as detailed in the Methods section. (**c**) Particle candidates are automatically determined by thresholding, and each candidate is checked for focusing criteria. The insets show the peak phase vs. focusing depth values (plotted in green) of 3 physical particles (color-coded arrows: red, orange and green) and 3 noise grains (purple arrows), all verified by SEM images, as illustrated in (**d**), following the same color coding as the arrows in (**c**). The best fit parabolas (plotted in yellow) are used to assess focusing criteria (see the Methods sections): the coefficient of the quadratic term for the bottom-right noise grain is significantly larger than the threshold and the R-squared goodness of fit values for the top and bottom-left noise grains do not satisfy the threshold unlike the physical/real particles, which satisfy these criteria. (**d**) We show the remaining physical particles after checking the focusing criteria for each particle candidate in (**c**). The insets show the SEM images of the detected particles as well as the empty regions co-localized with the noise grains (numbered and outlined in purple). The smallest particle detected is 26.4 nm in diameter, denoted with the red arrow and red image frame in (**c**) and (**d**), respectively. The color coded arrows point to these particles across (**a**),(**b**) and (**c**).

The majority of these remaining noise grains can be further smeared out by averaging the background compensated phase images of the same FOV that are reconstructed at different heights, around a neighborhood of the sample height (i.e., 20 *μm* with intervals of 1 *μm*) – see Fig. 5. It is observed that at each different reconstruction height, physical particles focus or de-focus but remain in the same lateral position at the corresponding reconstruction plane, while the noise grains move in random lateral directions. The resulting average image (Fig. 5d) strongly highlights the signatures of the physical particles, while further suppressing noise features. However some of the remaining noise grains could also generate particle-like lateral features if they happen to randomly overlap during averaging (see e.g., Fig. 6b and Supplementary Fig. S1). To mitigate this, each such particle candidate (determined through thresholding of the averaged phase image) is also tested for depth focusing: the physical particles focus, but the noise does not^29^. This additional depth-focusing criterion further helps to reject noise features and avoids false positives as illustrated in Fig. 6. Using this process, we were able to image polystyrene nanoparticles (size distribution provided in Supplementary Fig. S2) down to 26-nm diameter, which was independently verified by SEM (Fig. 6). The size of such particles is smaller than λ/10 and to our knowledge this constitutes the smallest particle detected using an on-chip microscope to date.

**Figure 7:**
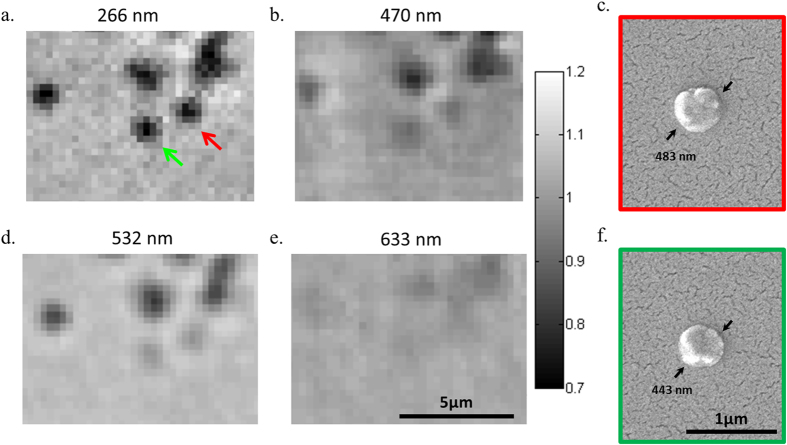
(**a**) SOD1 protein aggregates show significantly enhanced contrast in the amplitude reconstruction, imaged using the on-chip microscopy platform operating at 266 nm. (**b**),(**d**) and (**e**) The same aggregates imaged using a visible on-chip microscopy platform under blue (470 nm), green (532 nm) and red (633 nm) illumination light, respectively, all of which provide substantially lower contrast; also see Fig. 8. To provide a fair comparison we used the same number of frames, i.e., 556 frames, in panels a, b, d and e. The color bar represents the relative signal intensities in the holographic reconstructions. (**c**) and (**f**) SEM images of two representative protein aggregates of 483-nm (red arrow in (**a**)) and 443-nm (green arrow in (**a**)) in diameters.

**Figure 8:**
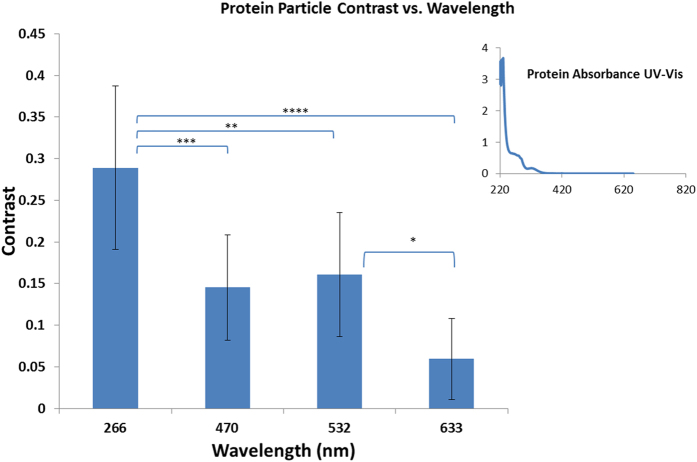
A quantitative comparison of the contrast measured for ten similar-sized (450–750 nm) SOD1 aggregates. The inset shows that the absorption of the SOD1 protein is significantly higher at 266 nm compared to the visible region, explaining the significantly higher contrast in the corresponding images from the UV on-chip microscopy platform. The P values were P < 0.1 for *, P < 0.01 for **, P < 0.001 for *** and P < 0.0001 for ****.

In addition to being able to detect smaller particles compared to visible illumination wavelengths, another advantage of using UV illumination is the increased absorption by biomolecules such as proteins and nucleic acids^33^,^34^. This enables exploiting the intrinsic absorption properties of biomolecules to image them with high sensitivity and contrast using our on-chip imaging platform operating at a 266-nm wavelength. As a proof of concept, we imaged sub-micron protein aggregates, which are a topic of high interest in biomedicine because they are linked to many neurodegenerative diseases and other proteinopathies^6^,^7^. For these proof-of-concept experiments, we used SOD1—an enzyme that is normally responsible for protection against superoxide radical anions formed during respiration. Aggregation of mutant SOD1 has been linked to amyotrophic lateral sclerosis (ALS), a fatal disease caused by the death of motor neurons^46^,^47^,^48^. Figure 7a shows the amplitude reconstruction image of SOD1 aggregates under 266-nm UV illumination, using on-chip microscopy with a very high contrast between the protein aggregates and the background signal. The same sample was also imaged by an on-chip microscopy platform^20^ under red (633-nm, Fig. 7e), green (532-nm, Fig. 7d) and blue (470-nm, Fig. 7b) illumination wavelengths using the same number of frames (i.e., 556) for frame averaging and pixel super-resolution. Compared to visible wavelengths, the contrast of the SOD1 aggregates is substantially higher with 266 nm illumination (see Fig. 8), highlighting the unique advantage of our UV on-chip microscopy platform in imaging biomolecules. The contrast values reported in Fig. 8 were calculated over ten individual SOD1 aggregates by measuring the difference between the average background signal and the minimum amplitude across the target aggregate and dividing this difference by the average background signal^57^. The significantly higher contrast (Fig. 8) under 266-nm illumination can be attributed to the high absorption of SOD1 in the UV part of the spectrum (see Fig. 8 inset), compared to the visible spectral region.

Both in the imaging of biomolecules, such as SOD1 aggregates, and nanoparticles, we have demonstrated how UV illumination helps on-chip holographic imaging reach new frontiers. Our platform benefits from the increased scattering and higher absorption properties of UV illumination, enabling us to decrease the nanoparticle detection limit of the on-chip imaging modality and also image biomolecules with significantly increased contrast. Using this UV wavelength required building a custom-designed free-space light delivery system composed of multiple UV lenses and other components. We believe that emerging LED technologies will make high-power and narrowband LEDs operating at UV wavelengths commercially available in the near future, further simplifying the illumination front-end unit of our current microscopy set-up.

## Methods

### UV On-chip Imaging Platform

Our on-chip microscopy platform (Fig. 1) is composed of a 4^th^ harmonic, Q-switched, Nd:YAG laser (FQSS 266-Q2, CyLaS GmbH, Berlin, Germany) a 13 Mega-pixel, 1.12 μm pixel pitch CMOS image sensor (IMX135, Sony Corporation, Tokyo, Japan) and a free-space light delivery system composed of UV lenses and motor controlled scanning galvo mirrors (GVS411, ThorLabs Inc., NJ, USA). The first stage of this light delivery system is composed of a spatial filter, which focuses the laser beam (f = 18 mm, Edmund Optics, NJ, USA) through a high-power 25 μm pinhole (Lenox Laser, MD, USA). The cleaned-up beam is then expanded by a factor of 10 (BE10-266, ThorLabs Inc., NJ, USA) so that the beam diameter would be sufficient to cover the full sensor active area of ~16.45 mm^2^. The beam is then reflected towards the first scanning galvo mirror from a harmonic beamsplitter (HBSY134, ThorLabs Inc., NJ, USA) that reflects 266 nm but transmits 532 nm and 1064 nm light to remove the residual 2^nd^ harmonic 532 nm and the base wavelength of 1064 nm light escaping from the laser. The spectrally and spatially cleaned up 266 nm pulse then reflects from the first scanning galvo mirror and passes through a 4-f lens system composed of a pair of UV lenses (f = 100 mm, Edmund Optics, NJ, USA) onto the second scanning galvo mirror which is placed at an orthogonal axis of rotation. From this second galvo, the beam is reflected through another identical 4-f system, towards the sensor placed at the focal plane of the final lens. The two scanning galvo mirrors and 4-f systems enable us to steer the incidence angle of beam in both axes across the image sensor while keeping the illuminated area constant on the image sensor. Both galvo mirrors were interfaced to a PC, through an I/O board (National Instruments, TX, USA) and controlled through a custom developed LabVIEW program, which automated the image acquisition and the positioning of the mirrors throughout each experiment.

### Sample Preparation

Because the 266-nm light is highly absorbed in regular glass slides, fused silica slides (10 mm-by-10 mm, 0.2 mm thick) that are transparent at this wavelength were used (MTI Corp., CA, USA). A handheld plasma generator was used to plasma-treat these slides for 1–2 minutes, making them hydrophilic, which is important for both sample binding and for the formation of self-assembled nanolenses. A ~3-μl droplet of the nanoparticle or protein aggregate solution was then deposited onto the plasma-treated side of the slide. After waiting ~3 minutes, the slide was carefully tilted allowing the remaining solution to drain. The slide was then gently washed with DI water to remove any excessive particles from the sample. After the UV imaging was completed, the samples used for SEM (Nova 600 NanoLab, FEI, OR, USA) imaging were further coated with 13.6 nm of gold by ion sputtering. This thickness of the gold coating was subtracted when reporting size measurements of the nanoparticles based on SEM images^28^.

### Sample Imaging and Nanolens Deposition

The image acquisition was fully automated using a custom-developed LabVIEW program, which adjusted the new galvo mirror positions for each frame. The first 196 images contained sub-pixel shifts of ~0.16 μm on a rectangular grid, and the remaining 360 images were shifted on a circular grid composed of 12 concentric circles with radial intervals of ~0.3 μm and 30 images per circle with equal angular spacing of 12°. To ensure reproducibility across experiments and account for any differences in the sample thickness, a *one-time* calibration step was introduced. Three images with predefined galvo mirror positions (ranging up to 0.2° tilt in the mirrors) were captured and the corresponding shifts in the image was calculated based on a visible mark (dust or any large object) located on the sample plane. The calibrated shift table, containing the angular positions for the two mirrors for the 556 images, was generated accordingly and input into the LabVIEW program.

For samples requiring nanolens deposition, the UV fused silica slides were first inserted into a portable vapor-deposition device^29^ where a reservoir of PEG (molecular weight: ~300 Da) was heated up to 90–100 °C and the vapor was channeled towards the sample. The deposition process was constantly monitored with the CMOS camera attachment of the same device, tracking the increase in the brightness of the holograms originating from larger objects in the FOV to assess the optimal time point for PEG deposition, which was observed to be around 5 min. Once the nanolens deposition was complete, the sample slide was carefully and rapidly transferred to the UV imaging platform and imaged within ~15 min, ensuring nanolens stability throughout the experiment.

### Pixel Super-Resolution with Circular Averaging and Image Reconstruction

The green channel of the image sensor was the most responsive to the 266-nm light and this channel was used to fill in the red and blue pixels of the image by averaging the neighboring green pixels^58^ in all the raw images. These raw frames were then background compensated (not to be confused with the background compensation of the phase images for nanoparticle detection) by passing them through a low-pass filter and dividing the initial frames with the filtered ones which eliminates the modulation in the background illumination. All these frames, which included 196 sub-pixel shifted images for pixel super-resolution and 360 circularly shifted images for circular averaging, were used to generate a noise-suppressed high-resolution image by a shift-and-add pixel super-resolution algorithm (Fig. 2)^52^,^53^. The algorithm first estimates the shifts in each frame, using the signatures of visible objects (e.g., dust or a mark) on the sample plane for image registration. This step is important for noise suppression since once the shift-and-add algorithm locks on to the holograms emanating from the sample plane, shifting and adding the low resolution images smears all the out-of-plane speckle and multiple reflection interference related noise artifacts, significantly reducing the background noise (Fig. 2). This shift estimation step is also important to account for any positioning errors in the galvo mirrors that might have occurred during the image capture, increasing the tolerance and robustness of the entire imaging platform.

The resulting noise-suppressed high-resolution image, with an effective pixel pitch of 0.28 μm, can then be digitally back-propagated using the angular spectrum approach^59^ to the correct object height (z_2_), reconstructing both the amplitude and phase images of the sample. For particle detection, the optimal z_2_ is defined as the height where the particle reconstruction shows minimum amplitude or maximum phase^28^,^29^. Other image sharpness features could also be used for reconstructing larger objects^20^,^60^.

### Digital Object Peeling and Twin-Image Elimination

If there are any large particles within the target FOV, the corresponding twin-image artifact of these larger particles could bury the signatures of the smaller, target nanoparticles. In this case, the high-resolution raw image is first forward-propagated to the twin-image plane (-z_2_) and the reconstructions of the larger objects (determined through simple thresholding of the phase image) are peeled off by replacing the object amplitude and phase with the background amplitude and background phase^29^. The resulting complex image after peeling is then back propagated to the sensor plane and saved as the new twin-image free raw image (now also a complex field) for further processing. In addition to their twin-images, these larger objects could also be digitally removed from the object plane in a similar manner. For example, in Fig. 6a and b, the larger particles were not removed from the object plane to help with image registration and comparison. However, they were removed in Fig. 6c and d to only highlight the smaller nanoparticles in the FOV.

### Focusing Behavior Check

The final step to distinguish the physical particles from noise grains and other spatial artifacts was to check the focusing behavior of these particle candidates where the reasoning is that the true particles focus whereas the noise features do not^29^. For this purpose, the particle candidates from the final averaged phase image were determined by simple thresholding. The location information of the particles was then used to extract the original peak-phase signal of each candidate across a z_2_ range of ±40 μm around the optimal z_2_ (i.e., z_2_^*^) with a step size of 2-μm. A parabola was fitted to the phase data of each candidate (Fig. 6c), and the focusing characteristic was determined based on the following properties of the fitted curve^29^: (1) the coefficient of the quadratic term should be <−9 × 10^−6^, (2) the R-squared goodness of fit should be >0.42, and (3) the peak of the parabola should be within a ±15 μm distance of the optimal z_2_ of each particle candidate. Particles that satisfy all these 3 criteria were kept as true particles, and all the rest of the particles were digitally cleaned, as detailed in the previous section (see e.g., Fig. 6d).

### SOD1 Aggregation and Absorbance Measurements

Wild-type SOD1 plasmid was used to transform the EG118 strain of *Saccharomyces cerevisiae* to produce the target protein, which was purified as described previously^61^. The demetallated (apo) SOD1 was prepared by dialyzing first against 50 mM ethylenediaminetetraacetic acid (EDTA) and 100 mM sodium acetate, pH 3.8, then against 100 mM NaCl and 100 mM sodium acetate, pH 3.8, and finally against 10 mM potassium phosphate (pH 7.0). The protein concentration was determined by using an extinction coefficient of 1.08 × 10^4^ M^−1^cm^−1^ 62. The purified SOD1 was diluted to 50 μM using 10 mM potassium phosphate, pH 7.0. The UV-visible spectrum of the protein solution was recorded using a quartz cuvette using a Beckman Coulter spectrophotometer.

To monitor the aggregation of apo-SOD1, the protein was dissolved at 50 μM in 10 mM potassium phosphate, 25 mM dithiothreitol (DTT) and 40 μM of ThT, pH 7.0 as described previously^48^. After mixing, 100 μL of the solution was placed in a 96-well plate and incubated at 37 °C with continuous shaking at ~1000 rpm for 48 h. The progress of aggregation was monitored by measuring ThT fluorescence every 2 h with λ_ex_ = 420 nm and λ_em_ = 485 nm using a Synergy HT plate-reader (BioTek Instruments, Inc., VT, USA). The SOD1 samples for UV holographic imaging were prepared simultaneously in solutions that did not contain ThT. The samples used for imaging were extracted at 19 h into this aggregation process.

## Conclusions

We have designed and built an on-chip holographic microscope operating at a UV wavelength of 266 nm, along with its custom-designed free-space light delivery system. Due to the high temporal and spatial coherence of the light source, speckle and other interference patterns have increased the background noise in the captured images, which was suppressed by capturing redundant circularly shifted images and averaging them via the pixel SR framework. With this circular averaging, our UV on-chip imaging platform detected 200 nm beads without the need for any nanolenses, in agreement with our RCS analysis. To detect smaller nanoparticles, further digital filtering steps, involving background compensation of the phase reconstructed images at different heights and averaging, were used in addition to nanolens deposition. Based on these, our on-chip imaging platform was able to detect nanoparticles as small as 26.4 nm. The platform was further tested for imaging SOD1 protein aggregates, which showed significant improvements in contrast compared to visible-range on-chip microscopy. We believe that future advances in LED technology could help simplify the illumination front-end of our current UV microscopy set-up, making it a broadly applicable platform for high-throughput imaging of biomolecules and nanoparticles.

## Additional Information

**How to cite this article:** Daloglu, M. U. *et al*. Computational On-Chip Imaging of Nanoparticles and Biomolecules using Ultraviolet Light. *Sci. Rep.* **7**, 44157; doi: 10.1038/srep44157 (2017).

**Publisher's note:** Springer Nature remains neutral with regard to jurisdictional claims in published maps and institutional affiliations.

## Supplementary Material

Supplementary Information
