# Supplementary material for: Computational On-Chip Imaging of Nanoparticles and Biomolecules using Ultraviolet Light
Source: Sci Rep. 2017 Mar 9;7:44157. doi: 10.1038/srep44157 (PMC5343455; doi:10.1038/srep44157)
Supplement: Supplementary Information [file srep44157-s1.pdf]

# Supplementary Information for

# Computational On-Chip Imaging of Nanoparticles and Biomolecules using Ultraviolet Light

**Authors:** Mustafa Ugur Daloglu[1,2,3], Aniruddha Ray[1,2,3], Zoltan Gorocs[1,2,3], Matthew Xiong[1], Ravinder Malik[4], Gal Bitan[4,5,6], Euan McLeod[7] and Aydogan Ozcan[1,2,3,8,*]

**Affiliations:**

[1]Electrical Engineering Department, University of California, Los Angeles, CA, 90095, USA.

[2]Bioengineering Department, University of California, Los Angeles, CA, 90095, USA.

[3]California NanoSystems Institute (CNSI), University of California, Los Angeles, CA, 90095, USA.

[4]Department of Neurology, David Geffen School of Medicine, University of California, Los Angeles, CA, 90095, USA.

[5]Brain Research Institute, University of California, Los Angeles, CA, 90095, USA.

[6]Molecular Biology Institute, University of California, Los Angeles, CA, 90095, USA.

[7]College of Optical Sciences, University of Arizona, Tucson, AZ 85721, USA

[8]Department of Surgery, David Geffen School of Medicine, University of California, Los Angeles, CA, 90095, USA.

*Correspondence: Prof. Aydogan Ozcan

E-mail: ozcan@ucla.edu

## Supplementary Figures:

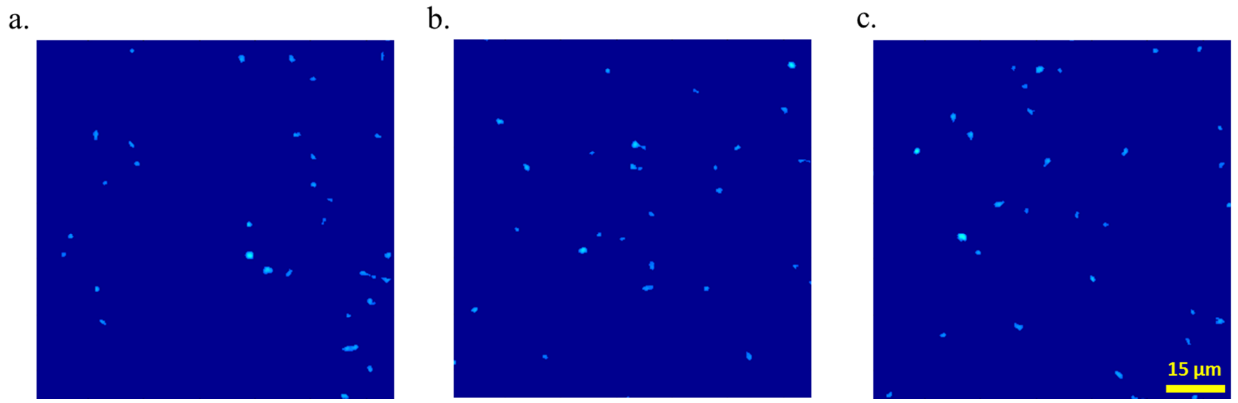


**Supplementary Figure S1**. **(a)**, **(b)** and **(c)** The noise grains removed from the reconstructions of three different samples with increasing nanoparticle densities: ~474 per $mm^2$ for (a), 1778 per $mm^2$ for (b) and 3082 per $mm^2$ for (c). The density of the removed noise grains in these images remains relatively constant: 4031 per $mm^2$ for (a), 3556 per $mm^2$ for (b), and 4098 per $mm^2$ for (c), despite the increasing nanoparticle density.

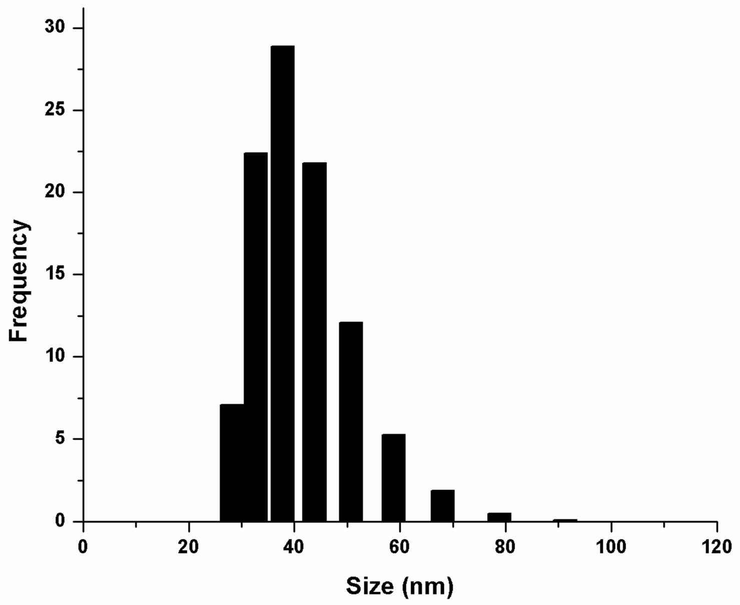


**Supplementary Figure S2.** The size distribution of the polystyrene nano-beads imaged with the help of the self-assembled nanolenses (see Fig. 6 of main text). Dynamic light scattering (ZetaSizer, Malvern Instruments) was used to measure the size distribution.
